# High-precision lung cancer subtype diagnosis on imbalanced exosomal data via Exo-LCClassifier

**DOI:** 10.3389/fgene.2025.1583081

**Published:** 2025-04-30

**Authors:** Siyu Zhan, Hao Yu, Shuang Liu, Ke Qin, Lu Guo

**Affiliations:** ^1^ Institute of Intelligent Computing, University of Electronic Science and Technology of China, Chengdu, Sichuan, China; ^2^ Trusted Cloud Computing and Big Data Key Laboratory of Sichuan Province, Chengdu, Sichuan, China; ^3^ School of Optoelectronic Science and Engineering, University of Electronic Science and Technology of China, Chengdu, China; ^4^ Yingcai Experimental College, University of Electronic Science and Technology of China, Chengdu, China; ^5^ Department of Pulmonary and Critical Care Medicine, Sichuan Provincial People’s Hospital, University of Electronic Science and Technology of China, Chengdu, China

**Keywords:** lung cancer, gene expression, WGAN, imbalanced data, DESeq2, 1D CNN

## Abstract

**Background and objective:**

Gene expression analysis plays a critical role in lung cancer research, offering molecular feature-based diagnostic insights that are particularly effective in distinguishing lung cancer subtypes. However, the high dimensionality and inherent imbalance of gene expression data create significant challenges for accurate diagnosis. This study aims to address these challenges by proposing an innovative deep learning-based method for predicting lung cancer subtypes.

**Methods:**

We propose a method called Exo-LCClassifier, which integrates feature selection, one-dimensional convolutional neural networks (1D CNN), and an improved Wasserstein Generative Adversarial Network (WGAN). First, differential gene expression analysis was performed using DESeq2 to identify significantly expressed genes from both normal and tumor tissues. Next, the enhanced WGAN was applied to augment the dataset, addressing the issue of sample imbalance and increasing the diversity of effective samples. Finally, a 1D CNN was used to classify the balanced dataset, thereby improving the model’s diagnostic accuracy.

**Results:**

The proposed method was evaluated using five-fold cross-validation, achieving an average accuracy of 0.9766 ± 0.0070, precision of 0.9762 ± 0.0101, recall of 0.9827 ± 0.0050, and F1-score of 0.9793 ± 0.0068. On an external GEO lung cancer dataset, it also showed strong performance with an accuracy of 0.9588, precision of 0.9558, recall of 0.9678, and F1-score of 0.9616.

**Conclusion:**

This study addresses the critical challenge of imbalanced learning in lung cancer gene expression analysis through an innovative computational framework. Our solution integrates three advanced techniques: (1) DESeq2 for differential expression analysis, (2) WGAN for data augmentation, and (3) 1D CNN for feature learning and classification. The source codes are publicly available at: https://github.com/lanlinxxs/Exo-classifier.

## 1 Introduction

Previous studies on early lung cancer diagnosis have predominantly focused on computed tomography (CT) imaging (Atmakuru et al., 2024; Han et al., 2021). Recently, exosomes have gained significant attention due to their widespread presence in bodily fluids, exceptional stability, and rich biological content, including proteins, DNA, mRNA, and non-coding RNA (Travis, 2020). As exosomes can mirror the characteristics of tumor cells, they hold immense potential for non-invasive diagnostics (Alharbi and Vakanski, 2023). Compared to traditional imaging and tissue biopsy methods, the collection of exosomes is simple, safe, and highly reproducible, providing a non-invasive means to reflect the molecular characteristics of tumors. This gives exosomal gene expression a significant advantage in early lung cancer screening, particularly in detecting small tumors or asymptomatic patients at early stages (Liu et al., 2024; Xu and Lu, 2024a; Xu and Lu, 2024b). Exosomal detection technology demonstrates significant advantages over conventional CT imaging and tissue biopsy in early cancer diagnosis, particularly in terms of molecular sensitivity, clinical operability, and multidimensional information acquisition. At the molecular level, exosomes enable detection of early-stage lesions smaller than 1 cm in diameter and carry specific biomarkers including miR-21 and miR-155. From a clinical operational perspective, the procedure requires only 2–5 mL of bodily fluid, eliminating the invasiveness associated with biopsies and radiation exposure from CT scans, while costing merely one-third of CT examinations. Particularly noteworthy is that exosomal analysis can advance the cancer diagnostic window by an average of more than 10 months compared to CT imaging and can identify circulating tumor microclusters undetectable by conventional radiological methods.

In recent years, significant advancements in deep learning and machine learning have been made in analyzing lung cancer gene expression, particularly in classifying and diagnosing major subtypes of non-small cell lung cancer (NSCLC), such as adenocarcinoma (AC) and squamous cell carcinoma (SCC). For example, in 2020, Fei et al. combined feature selection algorithms, such as Monte Carlo feature selection and genetic algorithms, with support vector machines (SVMs) and deep neural networks (DNNs) to identify high-information gene features and optimize classifiers through incremental feature selection (Yuan et al., 2020). Also in 2020, Satoshi Takahashi et al. integrated multi-omics data and deep learning to enhance NSCLC prognosis prediction accuracy (achieving an AUC of up to 0.99) while identifying potential molecular biomarkers (Takahashi et al., 2020). In 2022, Liu et al. proposed a KL-divergence-based gene selection method and developed a deep neural network using Focal Loss as the loss function, effectively improving classification performance (Liu and Yao, 2022). Additionally, Negar Maleki et al. and Margarita Kirienko et al. made progress in lung cancer feature selection and classification by utilizing genetic algorithms combined with k-nearest neighbors (k-NN) and radiogenomic analysis, respectively (Maleki et al., 2021).

Despite these advances, many studies have focused on single-type lung cancer prediction, with limited research on the classification of different lung cancer subtypes. Furthermore, existing methods often assume balanced data distribution, limiting their effectiveness in handling imbalanced datasets. High-dimensional gene expression data also present challenges, including excessive redundancy and noise, which may lead to model overfitting and reduced classification performance. Moreover, the complex and nonlinear relationships between gene expressions make it difficult to extract disease-relevant features and suppress irrelevant ones. In recent years, Generative Adversarial Networks (GANs) have gained attention in cancer diagnostics due to their potential for augmenting small sample datasets (Sedigh et al., 2019). However, their applications have predominantly focused on image data, and their adaptability to high-dimensional features such as exosomal gene expression remains underexplored (Khan et al., 2023).

This study proposes a novel deep learning-based framework, Exo-LCClassifier, designed to improve the diagnostic accuracy of lung cancer subtyping using exosomal gene expression data. The proposed method innovatively integrates DESeq2 for biologically informed feature selection, a Wasserstein Generative Adversarial Network (WGAN) for data augmentation and class balancing, and a one-dimensional convolutional neural network (1D CNN) for robust classification. In the first stage, DESeq2 is applied to identify significantly differentially expressed genes between normal and tumor samples, effectively reducing data dimensionality and retaining informative biomarkers. Next, WGAN is utilized to generate realistic synthetic samples, addressing the problem of class imbalance and enhancing model generalization. Finally, 1D CNN is employed to extract hierarchical representations and accurately classify gene expression patterns. Compared to existing machine learning and deep learning approaches, Exo-LCClassifier demonstrates consistently superior performance across multiple metrics, including accuracy, recall, and F1 score. These results highlight the model’s potential as a powerful and non-invasive diagnostic tool for lung cancer subtyping. Furthermore, this study addresses a critical gap in the current literature by introducing a fully integrated, data-driven pipeline tailored for exosomal RNA-seq analysis in oncology.

## 2 Methods

**FIGURE 1:**
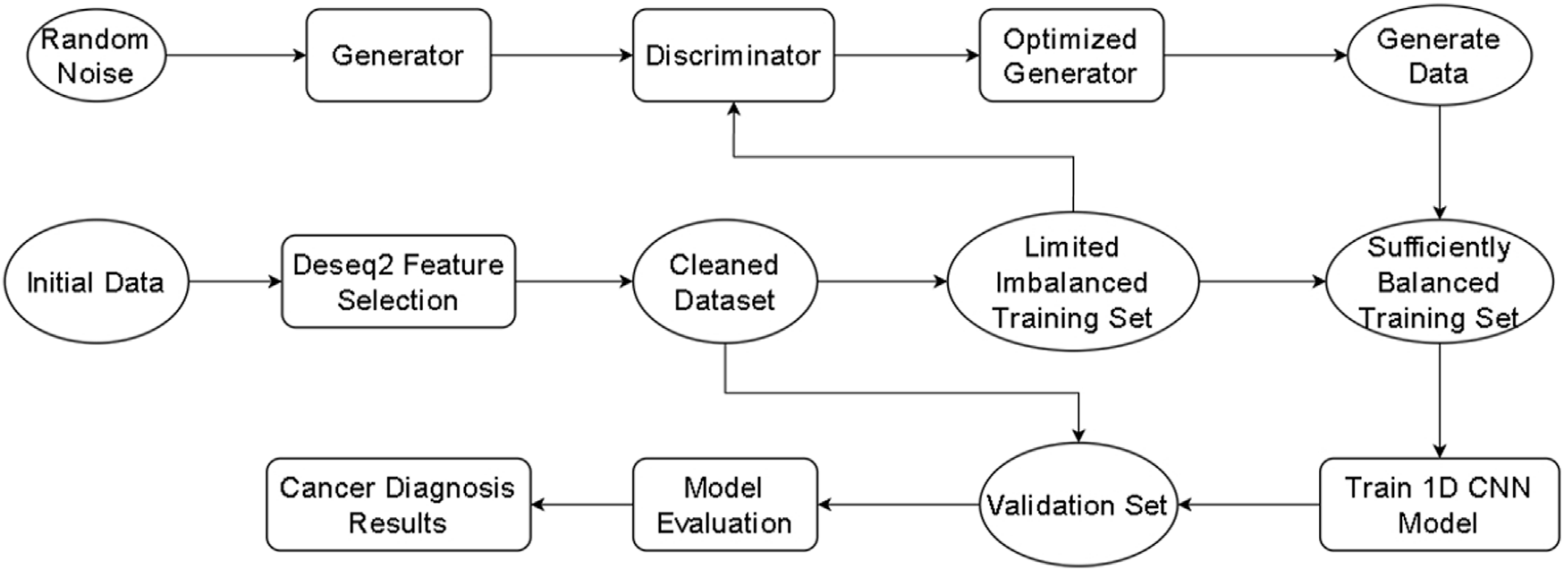
Exo-LCClassifier Architecture overview.

The proposed Exo-LCClassifier, follows the workflow shown in Figure 1. The process begins with differential gene expression analysis (DESeq2 (Love et al., 2014)) for data preprocessing, aimed at identifying significantly differentially expressed gene features between normal and tumor tissues, thereby simplifying high-dimensional data and extracting key information. Next, during the imbalanced learning phase, a Wasserstein Generative Adversarial Network (WGAN) is employed to generate new samples, balancing the distribution of the training dataset and effectively augmenting the minority class data. The balanced dataset is then used to train a custom-designed one-dimensional convolutional neural network (1D CNN). The model, after optimization, is capable of uncovering latent patterns in gene expression features and making accurate classifications. Finally, the trained 1D CNN model is applied to the test dataset to generate lung cancer diagnostic results. This method comprehensively addresses the challenges of high-dimensional data and class imbalance, providing a robust framework for accurate lung cancer diagnosis.

Each module of the Exo-LCClassifier was compared with similar modules from other methods to evaluate improvements at each stage. The differential expression analysis (DESeq2) used in Exo-LCClassifier was compared with other feature selection techniques (such as mutual information) to assess its ability to identify relevant gene features. The data imbalance handling module using WGAN in Exo-LCClassifier was compared with oversampling techniques like SMOTE and ADASYN from other methods to evaluate the effectiveness of the proposed method in balancing the data. Finally, the one-dimensional convolutional neural network (1D CNN) model in Exo-LCClassifier was compared with classification models used in other methods (such as decision trees and support vector machines) to highlight the advantage of deep learning in capturing complex patterns in gene expression data. Additionally, the final Exo-LCClassifier model was compared with three methods (NS-Forest, SPLR, and ZIPLDA) (Liu et al., 2023; Aevermann et al., 2021; Lambert, 1992), which were chosen as benchmarks due to their proven effectiveness in similar classification tasks.

### 2.1 Data collection and processing

In the data collection and processing phase, sample data were initially obtained from both The Cancer Genome Atlas (TCGA) and the Gene Expression Omnibus (GEO) databases. Specifically, data from the LUSC and LUAD projects within TCGA and additional lung cancer datasets from GEO were utilized. These samples contain RNA-seq-based gene expression profiles, with each sample representing a set of gene expression values. During the data extraction process, raw gene expression values were obtained from each sample and grouped accordingly. All samples were categorized into three groups: normal tissue, lung adenocarcinoma (LUAD), and squamous cell carcinoma (LUSC), facilitating subsequent analysis and comparison.

For data processing, normalization was applied to mitigate measurement discrepancies across different samples. This step ensured that gene expression values were comparable across all samples. Common normalization techniques, including log transformation and Z-score standardization, were used to effectively eliminate batch effects and systematic biases, thereby providing more consistent and reliable data for downstream analysis and model development.

### 2.2 DESeq2-based feature selection

The primary goal of feature selection in gene expression analysis is to identify the most relevant genes that differentiate between different tissue types, such as normal tissues and various cancer subtypes. This process reduces the dimensionality of the data, removing redundant or irrelevant features, which can improve the performance of machine learning models and simplify subsequent analyses.

With the rapid advancement of gene sequencing technology, the scale of gene expression data has expanded significantly. These data typically exhibit a high-dimensional structure, where each column represents a tissue sample, each row corresponds to a gene, and the feature values reflect gene expression levels. Despite the rich biological information contained in high-dimensional gene expression data, their analysis poses several challenges (Goveia et al., 2020).

On one hand, the high dimensionality often includes substantial redundancy and noise, which not only increases computational complexity but also reduces model robustness, potentially leading to overfitting and diminished classification performance (Wei et al., 2023). On the other hand, the limited number of samples exacerbates the issue of sparsity, further restricting the generalization capability of traditional machine learning models (Pes, 2020). Therefore, optimizing model performance under constrained sample conditions requires effective dimensionality reduction techniques.

Differential gene expression (DEG) analysis is an efficient feature selection method that identifies significantly differentially expressed genes (Muzellec et al., 2023).This approach filters out redundant information and selects critical features, providing refined inputs for model training, thereby improving classification accuracy and data adaptability (Bezuglov et al., 2023). In this study, we employed DESeq2 to perform differential expression analysis on gene expression data, identifying genes with significant differences among normal tissues, lung adenocarcinoma (LUAD), and squamous cell carcinoma (LUSC). Specifically, differential expression analysis was conducted on pairwise combinations of the three tissue types to identify gene features that exhibit significant differences between tissue groups.

DESeq2 identifies statistically significant differentially expressed genes (DEGs) by applying dual thresholds: an absolute log2 fold change (|log2FC| > 2) (indicating 2-fold differential expression) and Benjamini–Hochberg adjusted p-value <0.05. These stringent criteria effectively eliminate noise from random variations while retaining biologically meaningful signals. To construct a comprehensive feature set, we integrated DEGs identified in all pairwise comparisons (normal vs. LUAD, normal vs. LUSC, and LUAD vs. LUSC) through a union approach. This integrated feature space captures both common and subtype-specific molecular signatures, providing: enhanced discriminative power for lung cancer diagnosis, and a biologically interpretable foundation for subsequent classification modeling.

### 2.3 Adjusting imbalanced data using WGANs

To address the issue of data imbalance and enhance the model’s diagnostic performance on lung cancer exosome gene expression data, this study employs an improved Wasserstein Generative Adversarial Network (WGAN) for data augmentation. By generating synthetic data that closely resembles the real data distribution, WGAN effectively increases the training sample size, thereby improving the robustness of the classification model and enhancing its ability to distinguish between lung cancer subtypes.

Generative Adversarial Networks (GANs) are a type of deep learning architecture composed of two neural networks: a generator G and a discriminator D. These networks are trained in an adversarial manner to produce synthetic data that approximates the real data distribution. The generator G takes random noise z as input and generates fake samples G(z), while the discriminator D attempts to classify whether an input sample originates from the real data distribution or is generated by G.

During training, the generator aims to produce samples that are as realistic as possible to “fool” the discriminator, while the discriminator strives to accurately distinguish real data from generated data. The adversarial training objective is mathematically described in Equation 1:

$$\underset{G}{\min}\underset{D}{\max}\;V\left(D,G\right)=E_{x∼p_{\text{data}}}\left[\log D\left(x\right)\right]+E_{z∼p_{z}}\left[\log\left(1-D\left(G\left(z\right)\right)\right)\right]$$ (1)

In this context, pdata represents the distribution of real data, and pz denotes the noise distribution used as input to the generator. Through this adversarial process, the generator gradually produces samples that approximate the real data distribution. Wasserstein GAN (WGAN) improves upon traditional GANs by addressing their training instability. WGAN introduces the Wasserstein distance (also known as Earth Mover’s Distance) to measure the divergence between the real data distribution and the generated data distribution. This adjustment significantly enhances the quality of the generated samples (Weng, 2019). The loss function of WGAN is formulated as Equation 2:

$$\underset{G}{\min}\underset{D\in D}{\max}\;E_{x∼p_{\text{data}}}\left[D\left(x\right)\right]-E_{z∼p_{z}}\left[D\left(G\left(z\right)\right)\right]$$ (2)

In WGAN, the discriminator (or the critic D ) is constrained to be a 1-Lipschitz function, which is typically achieved through weight clipping. WGAN’s advantages lie in its smoother loss function, enabling the generator to receive more stable gradients, even when the sample distribution is imbalanced. This results in the generation of high-quality samples, which in turn improves the performance of classification models.

Traditional GANs and WGANs often employ convolutional neural networks (CNNs) as the primary architectures for both the generator and the discriminator, especially for tasks involving image data. However, the structural characteristics of gene expression data differ significantly from image data. Genes are not arranged in a fixed spatial relationship, and the correlations between genes are relatively low, which makes CNNs’ local receptive field capability less effective for gene expression data.

To address this, this study introduces tailored modifications to WGAN, replacing CNNs with deep fully connected neural networks (FCNNs) as the architectures for both the generator and discriminator. This adjustment better accommodates the unique characteristics of gene expression data. Furthermore, the model structure and parameters were optimized to enhance WGAN’s ability to handle imbalanced learning and improve classification performance on cancer gene expression datasets.

**FIGURE 2:**
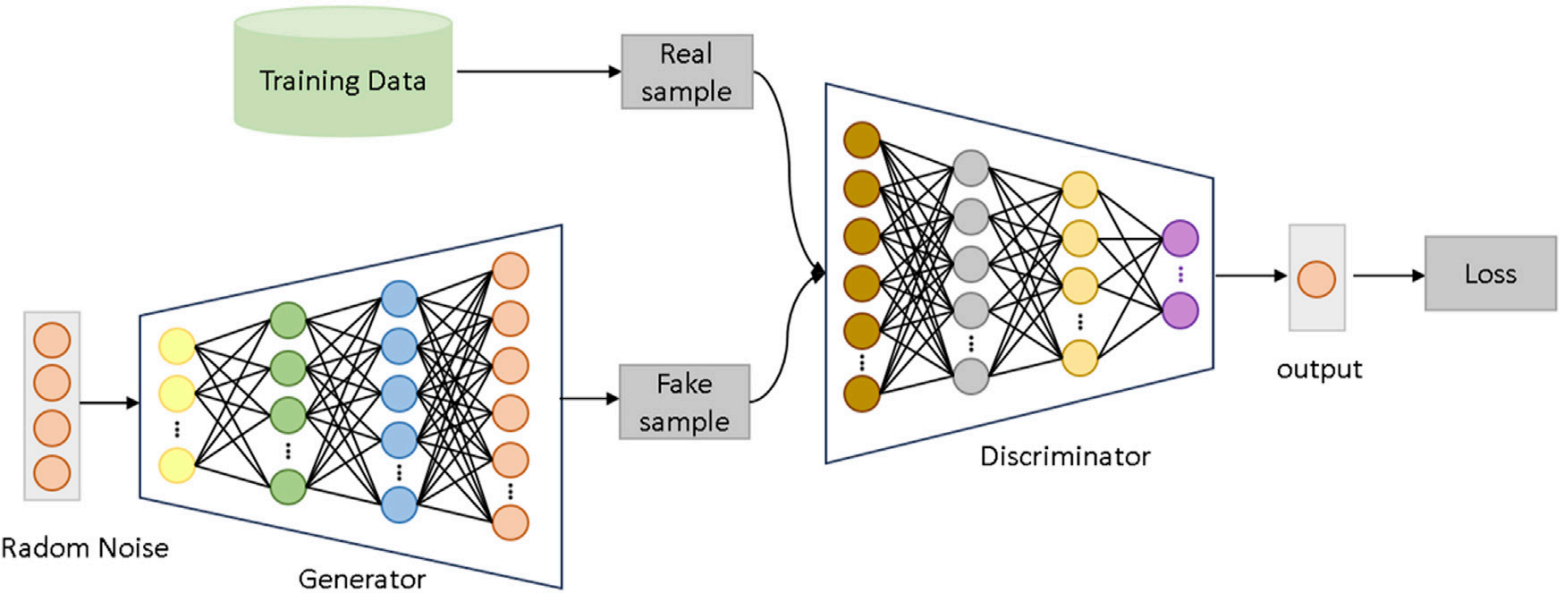
WGANs network architecture.

The improved Wasserstein Generative Adversarial Network (WGAN) used in this study consists of a generator and a discriminator, as shown in Figure 2. The generator receives random noise as input and aims to generate synthetic data that closely resembles the real gene expression data distribution. The discriminator, on the other hand, distinguishes whether the input data comes from the real distribution or the generator’s output. The network is structured with four layers for both the generator and discriminator, and the training is carried out with a learning rate of 0.0001, a batch size of 16, and a total of 200 epochs. The model also uses a clipping parameter of 0.01 to maintain the stability of the Wasserstein distance during training. Through adversarial training between the generator and the discriminator, the generator progressively produces synthetic samples that approximate the real data distribution, thereby enhancing the model’s diagnostic performance on lung cancer datasets.

This approach leverages the strengths of WGAN while addressing its limitations in handling non-image data, offering a robust framework for analyzing imbalanced cancer gene expression datasets.

In the generator network, random noise input is mapped to a high-dimensional gene expression feature space. The generator comprises multiple fully connected layers, employing the LeakyReLU activation function to enhance the model’s non-linear representation capability and generation quality. Specifically, the generator first maps 100-dimensional random noise to a 2960-dimensional feature space through a linear transformation. This output is then processed through two additional fully connected layers, which increase the model’s depth and complexity. The final output is a 2960-dimensional synthetic gene expression dataset.

The discriminator takes real or generated gene expression data as input. Its architecture similarly consists of multiple fully connected layers, with each layer followed by a LeakyReLU activation function. The discriminator’s objective is to continually optimize its classification ability to accurately distinguish real data from generated data. During training, the discriminator provides feedback to the generator through backpropagation, guiding it to produce more realistic data.

To ensure the stability and efficiency of the adversarial training process between the generator and the discriminator, RMSprop optimizer is used for parameter optimization, with the learning rate set to 0.0001. This optimization strategy effectively balances the convergence and stability of the model during training.

This network design enables the generator to produce gene expression data that closely resembles the real distribution. By significantly expanding the sample size, it addresses the data imbalance issue and further enhances the performance of the model in the diagnostic task for lung cancer exosome gene expression data.

### 2.4 Classification using 1D CNN

The purpose of employing a one-dimensional convolutional neural network (1D CNN) in this study is to leverage its ability to automatically extract meaningful features from high-dimensional gene expression data, thereby enhancing diagnostic accuracy and efficiency. By exploiting the latent patterns within the gene expression profiles, the 1D CNN enables a more effective classification of lung cancer subtypes, providing a robust model that can handle the complex and high-dimensional nature of genomic data.

In the classification stage, this study employed a one-dimensional convolutional neural network (1D CNN) as the classification model to fully exploit the latent features of gene expression data and enhance diagnostic performance. By applying convolution operations in a one-dimensional feature space, the 1D CNN effectively captures patterns and trends in gene expression levels while preserving the structural characteristics of the data.

Compared to traditional classification methods, 1D CNNs have the advantage of automatically extracting key features, reducing dimensionality, and minimizing redundant information (24, .). This approach significantly improves the model’s accuracy and efficiency, making it particularly suitable for analyzing high-dimensional and complex gene expression datasets.

**FIGURE 3:**
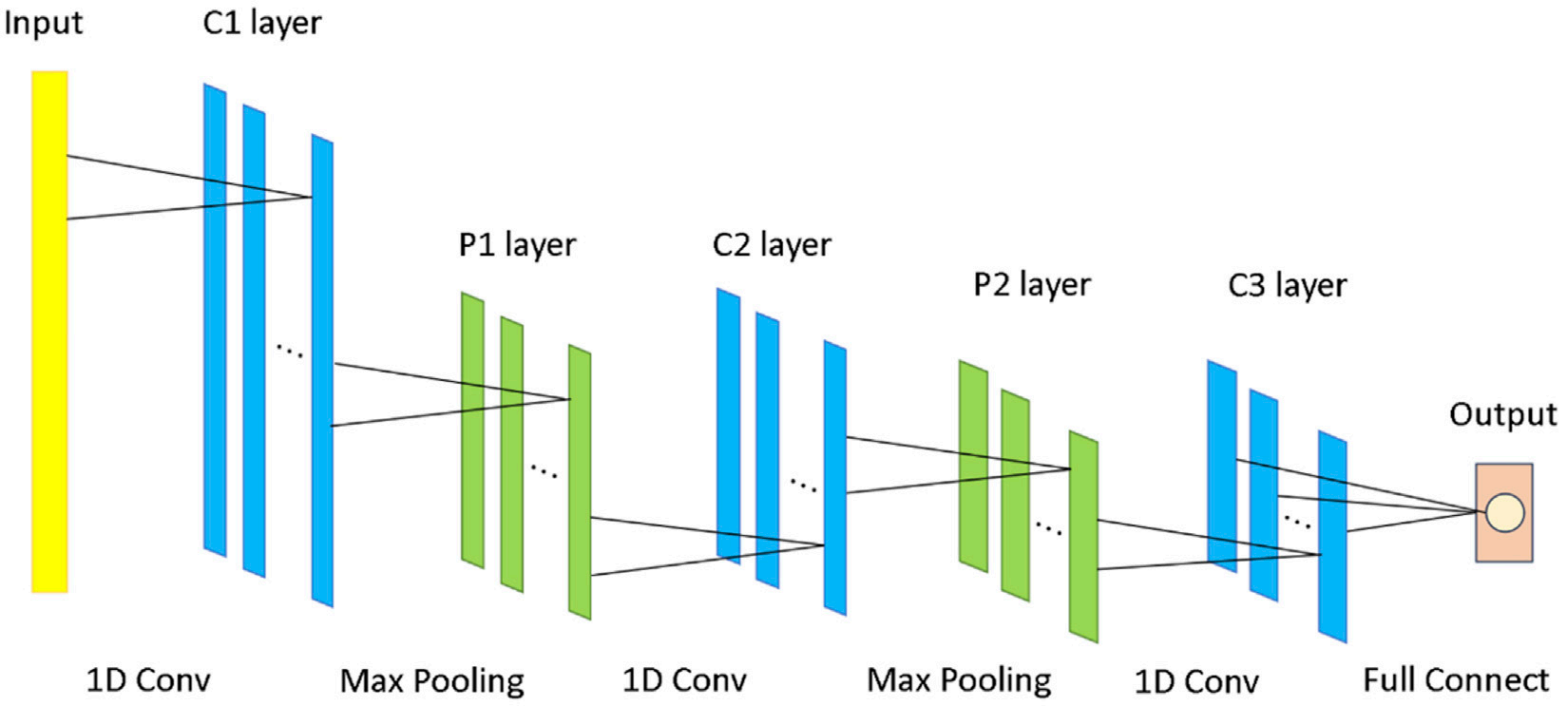
1D CNN network architecture.

The architecture of the 1D CNN consists of several key components aimed at maximizing feature extraction and classification performance, as illustrated in Figure 3. Initially, the input gene expression data passes through a one-dimensional convolutional layer with 16 output channels and three convolutional kernels of size 3. This layer is responsible for extracting local feature patterns from the input data. Next, a batch normalization layer is applied to normalize the features output by the convolutional layer, ensuring that the distributions of each layer’s outputs are similar, thus preventing issues like vanishing or exploding gradients. Subsequently, the ReLU activation function is used to introduce non-linearity, enhancing the network’s expressive power. A max-pooling layer is then added to downsample the convolutional feature maps, reducing the feature dimensions while retaining the most critical spatial information.

To further enhance the model’s focus on important feature regions, a spatial attention layer is incorporated. This layer automatically applies a weighted focus to the most critical areas for classification, thereby improving the quality of feature representation. To prevent overfitting and improve the model’s generalization ability, a dropout layer is added before the fully connected layer. This layer reduces model complexity by randomly dropping a portion of the neurons, thus improving the model’s performance on unseen data.

The network training process utilizes cross-entropy as the loss function and the Adam optimizer for parameter updates. The learning rate is set to 0.001, and the batch size is set to 16. To ensure the model’s generalizability, early stopping is applied to prevent overfitting to the training set. Additionally, hyperparameters such as learning rate and batch size are optimized through parameter selection via the validation set. Ultimately, after training and validating on preprocessed and balanced gene expression data, the 1D CNN establishes an optimized classification framework capable of distinguishing between different lung cancer subtypes, such as adenocarcinoma and squamous cell carcinoma. Experimental results demonstrate that the 1D CNN-based classification approach exhibits outstanding performance across various tissue types, providing robust support for accurate lung cancer subtype diagnosis.

### 2.5 Evaluation metrics

In this study, to comprehensively evaluate the performance of the proposed Exo-LCClassifier, we primarily use the following four evaluation metrics: accuracy, precision, recall, and F1-score. These metrics are calculated based on the confusion matrix. Accuracy refers to the proportion of correctly classified samples out of the total samples, as shown in Equation 3:

$$\text{Accuracy}=\frac{TP+TN}{TP+FP+FN+TN}$$ (3)

Precision quantifies the fraction of true positives among all predicted positives, as shown in Equation 4:

$$\text{Precision}=\frac{TP}{TP+FP}$$ (4)

Recall (Sensitivity) measures the fraction of true positives correctly identified, as defined in Equation 5:

$$\text{Recall}=\frac{TP}{TP+FN}$$ (5)

F1-score balances precision and recall via their harmonic mean, as formulated in Equation 6:

$$\text{F}1-\text{score}=2\times \frac{\text{Precision}\times \text{Recall}}{\text{Precision}+\text{Recall}}$$ (6)

In lung cancer diagnosis, accuracy, precision, recall, and F1-score are key metrics for evaluating model performance. Accuracy provides an overview of the model’s overall performance, reflecting the proportion of correct predictions across all samples. Precision focuses on evaluating the reliability of the model’s positive predictions, while recall measures the model’s sensitivity in identifying positive samples, directly influencing the detection rate of potential cases. In medical diagnostics, false negatives are particularly critical as they can cause patients to miss the optimal treatment window, making improving recall crucial. F1-score, as the harmonic mean of precision and recall, offers a more comprehensive evaluation for imbalanced datasets. By combining these metrics, the diagnostic model’s effectiveness can be more accurately assessed, providing a scientific basis for doctors and optimizing lung cancer screening and diagnostic decisions.

This suite of metrics, encompassing true positives, false positives, true negatives, and false negatives, thoroughly appraises the deep learning model’s capabilities in the realm of image semantic segmentation.

## 3 Experiments and results

### 3.1 Dataset

The dataset utilized in this study was derived from The Cancer Genome Atlas (TCGA) database, focusing on two major lung cancer projects: LUSC (lung squamous cell carcinoma) and LUAD (lung adenocarcinoma). The LUSC project included 553 samples, while the LUAD project comprised 600 samples, resulting in a combined dataset that offers comprehensive RNA-seq-based gene expression profiles. These datasets encompass 60,660 features representing gene expression levels. The samples were systematically categorized into three groups: normal tissue samples, lung adenocarcinoma samples, and squamous cell carcinoma samples. Additionally, data from the GEO (Gene Expression Omnibus) database were incorporated, which included 194 samples, further enhancing the dataset’s diversity and providing an external validation set for model testing. This combined dataset offers a robust foundation for the analysis and classification of lung cancer subtypes based on gene expression data.

### 3.2 1D CNN vs. baseline models

We evaluated the performance of our model against several traditional machine learning models, including Decision Tree (Quinlan, 1996), K-Nearest Neighbors (KNN) (Kramer and Kramer, 2013), Linear Discriminant Analysis (LDA) (Xanthopoulos et al., 2013), Naive Bayes (Webb et al., 2010), Random Forest (Rigatti, 2017), Support Vector Machine (SVM) (Hearst et al., 1998), and Logistic Regression (Nick and Campbell, 2007). According to the results in Table 1, we compared the performance of traditional models with the one-dimensional convolutional neural network (1D CNN) in lung cancer diagnosis. Among the traditional models, Linear Discriminant Analysis (LDA) achieved the best performance, demonstrating the highest or near-highest metrics in accuracy, precision, recall, and F1-score, with an accuracy of 0.9246. Random Forest also showed robust performance, maintaining consistently high results with an accuracy of 0.9081. Support Vector Machine (SVM) achieved an accuracy of 0.8925, with precision and F1-score comparable to those of LDA and Random Forest.

**TABLE 1:** Performance comparison of 1D-CNN and Classical models (with standard deviation).

Model	Accuracy	Precision	Recall	F1 score
Decision Tree	0.8959 ± 0.0153	0.8791 ± 0.0220	0.9090 ± 0.0135	0.8900 ± 0.0132
KNN	0.8222 ± 0.0587	0.7917 ± 0.0520	0.8677 ± 0.0395	0.8131 ± 0.0595
LDA	0.9246 ± 0.0230	0.9146 ± 0.0221	0.9437 ± 0.0174	0.9265 ± 0.0203
Naive Bayes	0.8847 ± 0.0213	0.9249 ± 0.0125	0.7427 ± 0.0318	0.7777 ± 0.0357
Random Forest	0.9081 ± 0.0225	0.9207 ± 0.0225	0.8999 ± 0.0313	0.9074 ± 0.0263
SVM	0.8925 ± 0.0238	0.9219 ± 0.0230	0.9134 ± 0.0129	0.9101 ± 0.0185
Logistic Regression	0.9055 ± 0.0304	0.8594 ± 0.0388	0.9311 ± 0.0226	0.8833 ± 0.0382
1D CNN	0.9306 ± 0.0242	0.8760 ± 0.1392	0.8888 ± 0.1187	0.8808 ± 0.1291

Other traditional models included Naive Bayes, which achieved an accuracy of 0.8847, demonstrating moderate performance across all metrics, and Decision Tree with an accuracy of 0.8959. K-Nearest Neighbors (KNN) achieved the lowest accuracy among the models, with an accuracy of 0.8222. In contrast, the 1D CNN achieved comparable or superior metrics across all evaluation criteria, with an accuracy of 0.9306, precision of 0.8760, recall of 0.8888, and F1-score of 0.8808, positioning it as a highly competitive model.

### 3.3 Feature selection results

Through feature selection, 2,960 genes were identified as inputs for classification and prediction. The results are visualized using a heatmap and a volcano plot, with a table summarizing the classification performance of various classifiers after feature selection.

**FIGURE 4:**
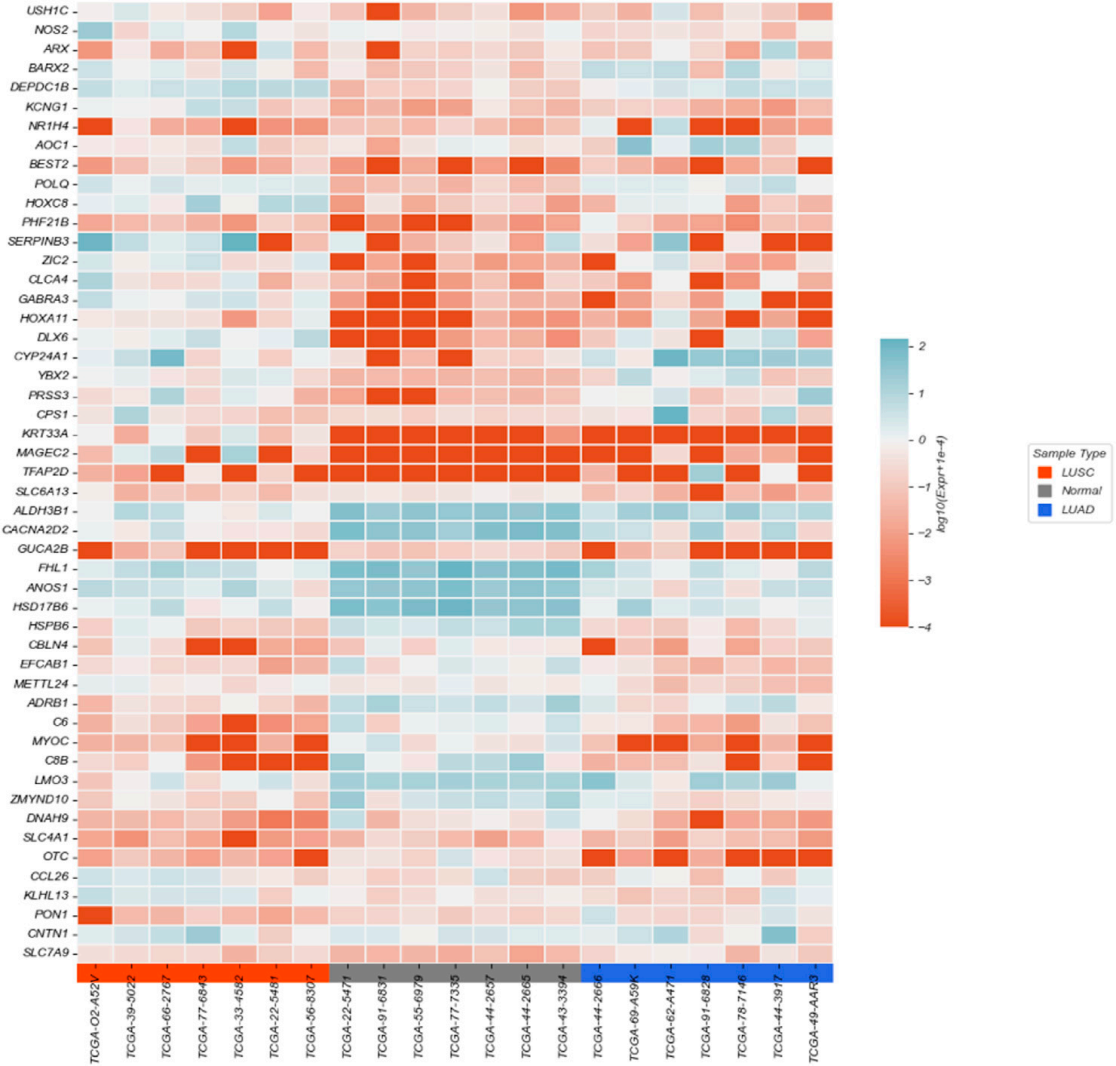
Heatmap of partial features.

The heatmap was constructed to illustrate the expression differences of 50 selected genes across 21 samples, including lung squamous cell carcinoma (LUSC), lung adenocarcinoma (LUAD), and adjacent normal tissues, as shown in Figure 4. The gene expression values were normalized and ${\text{log}}_{2}$ -transformed. Red indicates downregulation and blue indicates upregulation relative to the reference.

Samples are grouped by tissue type (LUSC in orange, LUAD in blue, and Normal in gray). The heatmap clearly shows distinct expression patterns among different tissue types, highlighting several genes with differential expression between tumor and normal tissues, as well as between cancer subtypes.

**FIGURE 5:**
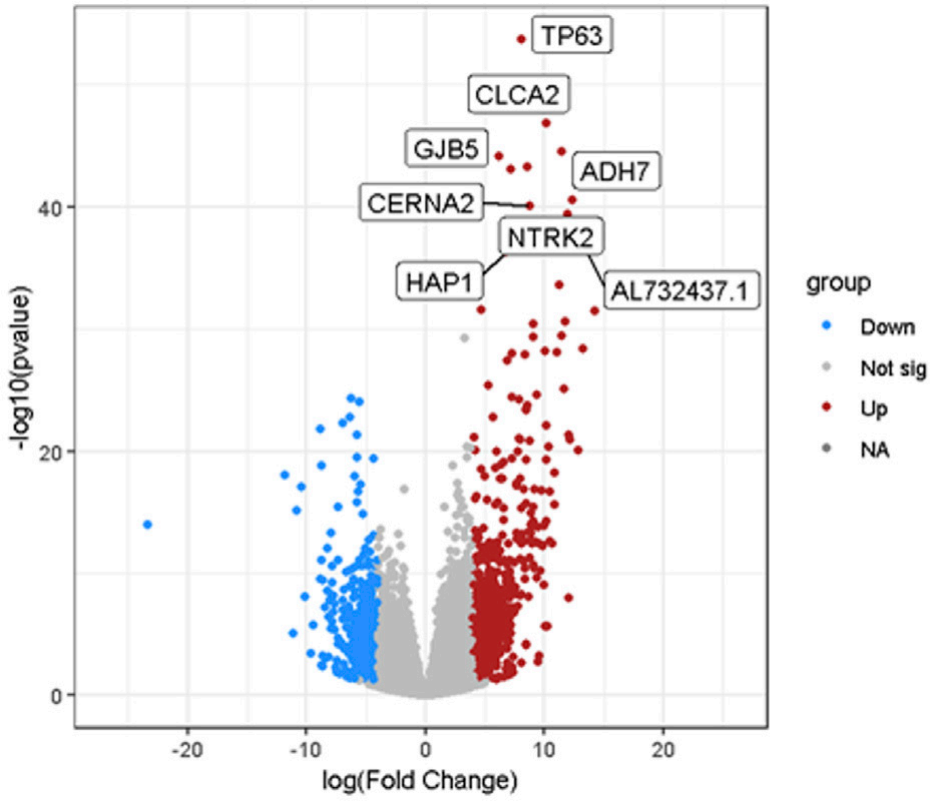
Volcano plot of features for cancer samples.

The volcano plot further reveals the differences in gene expression between the target and control groups, as shown in Figure 5. The horizontal axis represents the fold change in gene expression (log (Fold Change)), while the vertical axis indicates the significance level (-log10 (p-value)). Red dots denote significantly upregulated genes, such as TP63, NTRK2, and CLCA2, highlighting their elevated expression in cancer samples. Blue dots correspond to significantly downregulated genes, indicating reduced expression in cancer states, while gray dots represent genes that do not reach the significance threshold.

Table 2 presents a comparative performance evaluation of six feature selection methods (mean ± standard deviation). Notably, our adopted DESeq2 method demonstrates superior performance across multiple metrics: it achieves the highest accuracy (0.9532 ± 0.0138), recall (0.9651 ± 0.0104), and F1-score (0.9563 ± 0.0116) among all methods. This outstanding performance can be attributed to DESeq2’s negative binomial distribution model specifically designed for count data, which enables more accurate identification of differentially expressed features, particularly demonstrating significant advantages in processing biomedical data.

**TABLE 2:** Performance comparison of different feature selection methods.

Method	Accuracy	Precision	Recall	F1 score
F-test	0.9410 ± 0.0185	0.9413 ± 0.0196	0.9514 ± 0.0167	0.9450 ± 0.0166
Chi-squared Test	0.9454 ± 0.0141	0.9502 ± 0.0127	0.9547 ± 0.0121	0.9515 ± 0.0098
Mutual Information	0.9497 ± 0.0138	0.9511 ± 0.0088	0.9554 ± 0.0142	0.9523 ± 0.0097
Variance Threshold	0.9471 ± 0.0143	0.9533 ± 0.0166	0.9512 ± 0.0103	0.9515 ± 0.0111
ANOVA	0.9488 ± 0.0127	0.9492 ± 0.0124	0.9572 ± 0.0106	0.9518 ± 0.0086
DESeq2	0.9532 ± 0.0138	0.9491 ± 0.0133	0.9651 ± 0.0104	0.9563 ± 0.0116

While mutual information (accuracy: 0.9497 ± 0.0138) and ANOVA (F1-score: 0.9518 ± 0.0086) show acceptable performance, they remain inferior to DESeq2 in overall metrics. It is particularly noteworthy that DESeq2’s exceptional recall performance (0.8 percentage points higher than the suboptimal ANOVA method) indicates its remarkable effectiveness in reducing false negatives, which is crucial for ensuring comprehensive feature inclusion. Furthermore, all methods maintain relatively low standard deviations (<0.02), further validating the reliability of DESeq2’s results.

These comparative results substantiate the rationale for selecting DESeq2 as our feature selection method, and its excellent performance will provide more reliable feature sets for subsequent analyses.

### 3.4 WGANs performance

**FIGURE 6:**
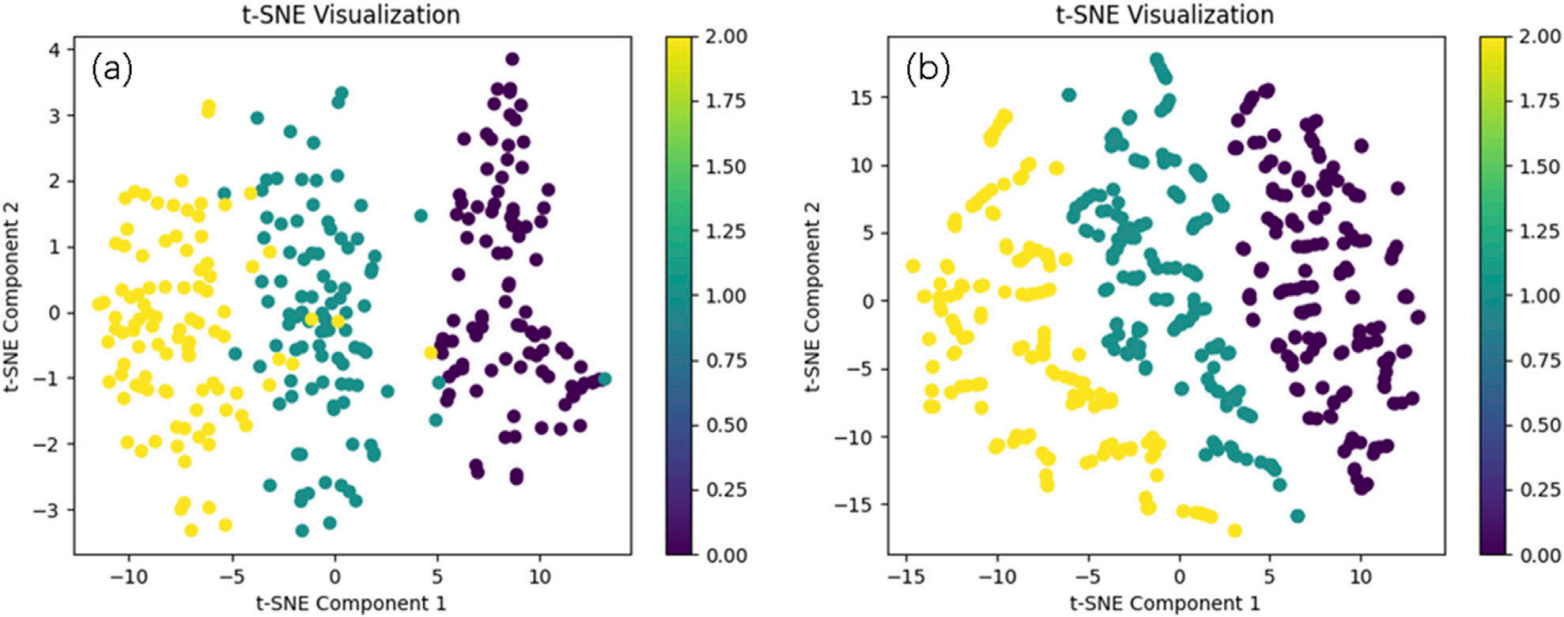
The real data distribution and the data distribution generated by WGANs. Comparison of Original Data Distribution **(a)** and Generated Data Distribution by WGANs **(b)**.

We present the visualized data of the original dataset after t-SNE dimensionality reduction, as well as the data generated by WGANs, as shown in Figure 6. (a) illustrates the distribution of high-dimensional features of the original data in a two-dimensional space, providing an intuitive representation of the clustering patterns between different samples. (b) shows the distribution changes after data expansion with WGANs. In the plot, we observe that the distribution of the WGANs-generated data is largely consistent with the original data.

Table 3 compares the performance of four data augmentation methods across key evaluation metrics (mean ± standard deviation). Our implemented WGAN approach demonstrates superior performance, achieving the highest scores in all metrics: accuracy (0.97659 ± 0.00703), precision (0.97624 ± 0.01005), recall (0.98274 ± 0.00503), and F1-score (0.97927 ± 0.00681).

**TABLE 3:** Performance comparison of data augmentation methods.

Method	Accuracy	Precision	Recall	F1 score
MIXUP	0.9575 ± 0.0148	0.9599 ± 0.0087	0.9660 ± 0.0101	0.9627 ± 0.0090
ADASYN	0.9593 ± 0.0133	0.9600 ± 0.0179	0.9700 ± 0.0099	0.9642 ± 0.0139
SMOTE	0.9610 ± 0.0142	0.9633 ± 0.0120	0.9708 ± 0.0110	0.9665 ± 0.0118
WGAN	0.9766 ± 0.0070	0.9762 ± 0.0101	0.9827 ± 0.0050	0.9793 ± 0.0068

The significant performance advantage of WGAN (approximately 1.5-2 percentage points higher than suboptimal methods) can be attributed to its adversarial training framework that generates more realistic synthetic samples, particularly effective for addressing class imbalance problems. Notably, WGAN’s exceptionally high recall (0.98274) indicates its outstanding capability in minimizing false negatives, which is crucial for sensitive applications.

While SMOTE shows competitive results (F1-score: 0.96648 ± 0.01179) among conventional methods, all non-WGAN approaches exhibit clear performance gaps. The consistently low standard deviations (<0.02) across all methods confirm the reliability of these comparative results.

### 3.5 Comparison of advanced methods

Table 4 presents the performance comparison of various feature selection and classification methods in terms of accuracy, precision, recall, and F1 score. Among all the evaluated models, the proposed Exo-Classifier achieved the highest overall performance, with an accuracy of 0.9766, precision of 0.9762, recall of 0.9827, and an F1 score of 0.9793. These results notably surpass those of the other advanced methods. Although NS-Forest and SPLR demonstrated competitive performance, with F1 scores of 0.9613 and 0.9594 respectively, and ZIPLDA achieved an F1 score of 0.9571, none matched the overall balance and robustness of the Exo-Classifier. These findings highlight the superior effectiveness of the proposed method in accurately classifying lung cancer subtypes based on exosomal gene expression data.

**TABLE 4:** Performance comparison of feature selection and classification methods.

Model	Accuracy	Precision	Recall	F1 score
NS-Forest	0.9540 ± 0.0139	0.9612 ± 0.0160	0.9631 ± 0.0088	0.9613 ± 0.0116
SPLR	0.9558 ± 0.0118	0.9559 ± 0.0163	0.9647 ± 0.0123	0.9594 ± 0.0122
ZIPLDA	0.9540 ± 0.0130	0.9508 ± 0.0198	0.9657 ± 0.0099	0.9571 ± 0.0148
Exo-Classifier	0.9766 ± 0.0070	0.9762 ± 0.0101	0.9827 ± 0.0050	0.9793 ± 0.0068

### 3.6 External validation with GEO data

Table 5 reports the performance of different deep learning strategies for lung cancer subtype classification, evaluated in terms of accuracy, precision, recall, and F1 score. The baseline 1D CNN model achieved an accuracy of 0.9072 and an F1 score of 0.9178, indicating reasonable performance. When combined with DESeq2 for feature selection, the performance improved substantially, yielding an accuracy of 0.9381 and an F1 score of 0.9381, demonstrating the effectiveness of integrating biologically informed feature selection. Notably, the proposed Exo-Classifier outperformed both baseline models, achieving the highest accuracy (0.9588), recall (0.9678), and F1 score (0.9616), highlighting its superior ability to capture informative patterns from exosomal gene expression data and its robustness in classifying lung cancer subtypes.

**TABLE 5:** Performance comparison of different deep learning strategies.

Model	Accuracy	Precision	Recall	F1 score
1D CNN	0.9072	0.9332	0.9142	0.9178
1D CNN + DESeq2	0.9381	0.9481	0.9315	0.9381
Exo-Classifier	0.9588	0.9558	0.9678	0.9616

## 4 Discussion

In comparison with traditional machine learning models, the 1D CNN demonstrates a clear advantage in lung cancer diagnosis. Although models like LDA, Random Forest, and Support Vector Machine also show good performance in terms of accuracy and other metrics, the 1D CNN outperforms all traditional models in terms of accuracy, precision, recall, and F1 score. The superior performance of the 1D CNN can be attributed to its ability to automatically extract important features from the data and perform nonlinear mappings through deep networks. This makes it particularly well-suited for learning from high-dimensional data and complex patterns, whereas traditional models rely on manually engineered features or assumptions, which are less effective in capturing the intricate relationships within the data.

Feature selection played a critical role in enhancing model performance by selecting genes with significant discriminative power. This process reduced the dimensionality of the data, lowered computational complexity, and eliminated redundant information, allowing the model to focus on the most informative features. As a result, the model was better able to capture key patterns related to lung cancer diagnosis, free from the interference of noise or irrelevant features, thus improving both accuracy and robustness. The effectiveness of feature selection was clearly illustrated through heatmap and volcano plot visualizations, which revealed significant differences in gene expression between normal and cancer samples. In particular, the heatmap showed distinct expression patterns in LUSC and LUAD samples, highlighting specific genes that are crucial for identifying the different lung cancer subtypes. The volcano plot further confirmed the importance of these genes by pinpointing significantly upregulated and downregulated genes, such as TP63, NTRK2, and CLCA2, which are strongly associated with cancer.

The performance improvements of various classifiers after feature selection further validate the effectiveness of our approach. The CNN model, in particular, showed notable gains in accuracy, precision, and F1 score, demonstrating that the optimized features made the model more sensitive to the data and improved its ability to learn and classify cancer samples. The significant increase in F1 score suggests that our method addressed the issue of imbalanced data, enhancing the classifier’s ability to avoid bias towards any one class. Traditional classifiers, such as KNN and SVM, also benefited from feature selection, showing improved accuracy and precision. This demonstrates that feature selection helps these models better adapt to high-dimensional data and enhances their classification ability. Overall, feature selection not only improved classification performance but also provided greater interpretability, helping us to focus on the most relevant features for lung cancer diagnosis.

Analyzing the t-SNE visualization of WGAN-generated data and classifier performance highlights the critical role of data augmentation in enhancing model accuracy. The t-SNE results demonstrate that the generated data closely aligns with the original dataset’s distribution, effectively capturing its key features while avoiding noise or bias. This consistency ensures a solid foundation for classification tasks, particularly in medical datasets where maintaining the authenticity of data distribution is essential.

The performance improvements across classifiers further validate the benefits of data augmentation. The 1D CNN model achieved the highest accuracy, precision, recall, and F1-score, indicating that the augmented data provided a richer and more diverse feature space for learning complex patterns. Traditional models like Random Forest and LDA also showed significant gains, underscoring the broad applicability of WGAN-generated data. These improvements can be attributed to increased sample size, better data representativeness, and improved handling of class imbalance, all of which enhance the robustness and reliability of machine learning models.

## 5 Conclusion

This study proposes a method named Exo-LCClassifier, which integrates multiple techniques to effectively address the challenges of class imbalance, high dimensionality, and noise in cancer gene expression data. We designed an improved oversampling strategy based on the Wasserstein Generative Adversarial Network (WGAN), utilizing a fully connected deep network structure to expand each class to 1,000 samples, thereby enhancing data diversity and representativeness. Additionally, we employed the DESeq2 feature selection method to identify significantly expressed cancer-related genes from high-dimensional data, reducing noise and improving model generalization. Based on this expanded dataset, we implemented a one-dimensional convolutional neural network (1D CNN) for classification tasks. The results demonstrate that our model exhibits outstanding performance in cancer subtype diagnosis, providing high accuracy and stable, reliable classification outcomes. By integrating deep learning with traditional statistical methods, this study not only enhances classification performance but also offers new insights into cancer gene expression analysis, contributing to both theoretical research and practical applications in cancer diagnosis and treatment.

## Data Availability

The data presented in this study are openly available in The Cancer Genome Atlas (TCGA) database (https://portal.gdc.cancer.gov/) under project IDs TCGA-LUSC and TCGA-LUAD, and in the Gene Expression Omnibus (GEO) database (https://www.ncbi.nlm.nih.gov/geo/) under accession number GSE81089.
